# Resident Knowledge and Perception of Pain Management

**DOI:** 10.7759/cureus.6107

**Published:** 2019-11-08

**Authors:** Jose Garcia, Levonti Ohanisian, Angel Sidley, Allison Ferris, George Luck, Garrett Basich, Abraham Garcia

**Affiliations:** 1 Internal Medicine, Charles E. Schmidt College of Medicine, Florida Atlantic University, Boca Raton, USA; 2 Orthopaedic Surgery, Charles E. Schmidt College of Medicine, Florida Atlantic University, Boca Raton, USA; 3 Biomedical Science, Charles E. Schmidt College of Medicine, Florida Atlantic University, Boca Raton, USA; 4 Integrated Medical Science, Charles E. Schmidt College of Medicine, Florida Atlantic University, Boca Raton, USA; 5 Medicine, St. Mary's College of California, Moraga, USA; 6 Microbiology, University of Florida, Gainesville, USA

**Keywords:** pain management, medical education, opioid education, residency education, pain curriculum, opioids

## Abstract

Chronic pain involves a complex mechanism that afflicts 50 million adults in the United States and incurs societal costs upwards of $560 billion annually. The consequences of this epidemic have resulted in an epidemic of its own, with the opioid crisis becoming a top priority in healthcare. Historically, the sub-optimal practices of overprescribing opioids and inadequate monitoring of iatrogenic addiction have contributed to this problem. If progress is to be made in this area, it is imperative that we examine how future physicians are being trained to manage pain. We examined internal medicine resident knowledge regarding pain as well as their satisfaction with medical school preparation in this regard using two surveys: The Knowledge and Attitudes Survey Regarding Pain (KASRP) and The Medical School Pain Curriculum Survey (MSPCS). Residents scored an overall 60.7% on the knowledge assessment survey, and less than 50% of respondents agreed that their medical school curriculum had prepared them sufficiently. This suggests that improvements can be made in medical school curricula regarding pain management education to better train physicians on how to manage pain, particularly in an era that demands expertise in this area.

## Introduction

Chronic pain is defined as pain lasting or recurring for more than three to six months [1]. It is one of the most common reasons adults seek medical care [2]. The prevalence of chronic pain ranges from 11% to 40% and is associated with multiple comorbid conditions, both physical and mental; additionally, those of lower socioeconomic status are afflicted disproportionately [3]. According to a 2016 report by the Centers for Disease Control (CDC), 20.4% of Americans had pain that met the definition of chronic pain [3]. Chronic pain not only has a significant impact on society financially, including healthcare and loss of productivity costs cumulating $560-635 billion annually but also presents significant morbidity across the population. Attempts to address chronic pain have unfortunately given way to a new epidemic of substance use disorder (SUD) and overdose [3-4]. In 2015, two million Americans had SUD involving prescription pain medications, with 20,101 accidental overdose deaths related to those medications [5]. In order to reduce the number of deaths, prescription misuse, and associated morbidity and mortality linked to prescription pain medication, many medical schools have prioritized re-evaluating and adapting their pain curriculum to address society’s changing needs [6-7]. Improving the medical graduates’ understanding of pain and SUDs should be part of a multifaceted strategy to minimize inappropriate prescribing of opioids. The AAMC recently released survey results from US medical schools demonstrating that 87% of responding colleges address all four domains reviewed: 1. the nature of pain; 2. pain assessment and measurement, including assessment of risk for SUD; 3. management of pain, including SUD treatment and opioid overdose; and 4. the context of pain and SUD [7]. Lectures, clinical experiences, and case-based learning were the most common methods used. The responding colleges reported the challenges of implementation to be lack of time in the curriculum, lack of faculty expertise, and difficulty of assessment.

Although many medical schools cover the four domains of pain, many do not assess the efficacy of their initiatives [7]. An appropriate time to assess competency in pain management, and, therefore, the efficacy of educational initiatives, is early in residency, once trainees begin to apply their knowledge. Although surveys on pain knowledge and attitudes have been used to assess physician, nurse, and pharmacist preparedness, to our knowledge, they have not been used on residents with the intent to highlight areas of improvement in both undergraduate and graduate medical education [4,8-9]. The purpose of this study was to examine internal medical resident knowledge regarding pain, in order to assess whether increased education in the formative years for physicians is necessary to help address the opioid crisis.

## Materials and methods

In order to evaluate knowledge and attitudes regarding medical school curricula, two distinct questionnaires were administered to first-year internal medicine residents. Since investigators felt residents could better self-reflect on their medical school curriculum after they had clinical experience, the surveys were administered two to three months after orientation. 

The first survey assessed residents’ perception of their undergraduate medical school curriculum in preparing them to manage patients in pain or at risk for SUDs. For this purpose, the investigators created the Medical School Pain Curriculum Survey (MSPCS). The MSPCS was created using an inter-disciplinary approach, incorporating topics that a focus group of internists, anesthesiologists, medical residents, and medical students indicated as important when preparing physicians to manage pain. The survey contained 31 questions utilizing a four-point Likert scale that addressed residents’ impressions regarding the strengths and weaknesses of pain education in medical school. 

The second survey assessed the knowledge regarding the assessment and management of pain, highlighting proficiencies and deficiencies, using an established assessment tool. We employed the Knowledge and Attitudes Survey Regarding Pain (KASRP), a tool developed by Ferrell and McCaffery in 1987 and updated by the same authors in 2012 to reflect the modern standards of care as per the World Health Organization, the American Pain Society, and the National Comprehensive Cancer Network Pain Guidelines [10]. The KASRP has been used to assess knowledge of pain management in healthcare professionals and students in the United States, United Kingdom, Israel, and Jordan [4]. To the best of our knowledge, the KASRP has never been used with residents.

Both surveys were distributed to 24 first-year internal medical residents at Florida Atlantic University (FAU). Responses were not only collected, scored, and stratified by individual respondent scores but also by individual questions and percent of respondents answering each question correctly.

## Results

KASRP

All 24 residents submitted responses to the KASRP, with 19 (79%) respondents answering every question. The overall KASRP performance was somewhat low, with an average correct response rate of 60.7% (SD 14.4%). The scores ranged from 31.7% to 89.3%. Each question was analyzed individually by the percent of respondents who answered correctly and divided into four topics: assessment of pain, pain management, pharmacology of pain, and substance use disorder. The average correct response rates for questions in each of these categories were 79%, 61%, 60%, and 67%, respectively.

Among assessment questions, one question incorrectly answered by over half of the respondents pertained to assessing post-operative pain. However, all respondents correctly answered the three questions on assessing pain in children.

Among the pain management questions, six questions were answered incorrectly by more than half of the respondents. These questions pertained to treating cancer-related pain, managing pain of unspecified origin, and dosing and administering opioids. All respondents correctly identified that treating severe, acute, traumatic pain requires intravenous medication.

Regarding the pharmacology of pain medications, six questions were answered incorrectly by more than half of the respondents. These questions focused on understanding the pharmacodynamics of opiates, identifying normal responses to opiates, and identifying patients who were at risk for adverse reactions such as respiratory depression. All respondents correctly answered questions on the pharmacodynamics of gabapentin, a non-opioid medication explicitly used for neuropathic pain.

In the domain of SUD, over half of the respondents overestimated the prevalence of pre-existing drug or alcohol abuse among patients who present with severe pain. Over half failed to characterize symptoms of abrupt opiate discontinuation in opioid-dependent patients correctly. All respondents failed to define narcotic addiction accurately.

MSPCS

**Figure 1:**
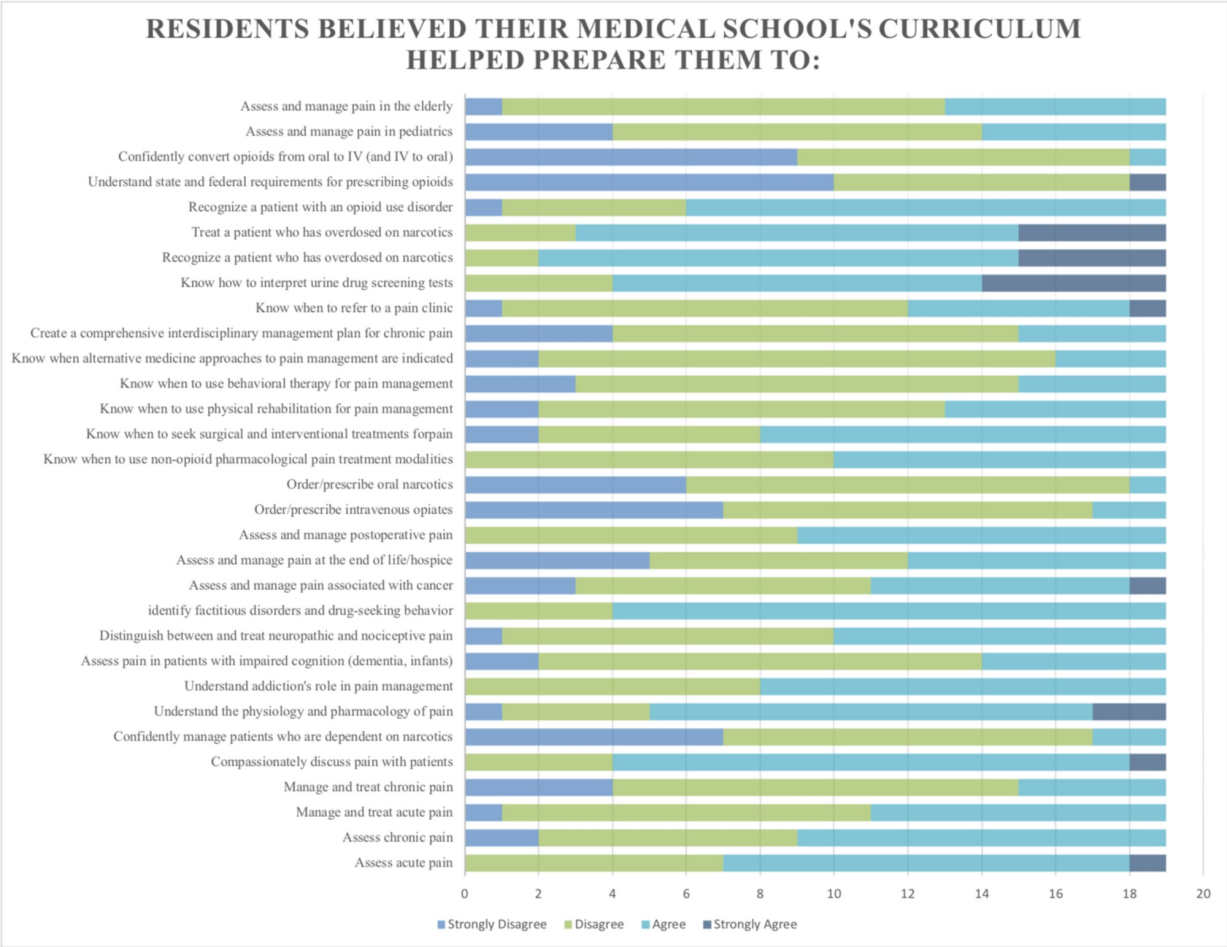
Results for the Medical School Pain Curriculum Survey (MSPCS) distributed to first-year internal medicine residents

A total of 19 residents (79%) completed the MSPCS. For the competencies addressed, 57.4% of respondents either disagreed or strongly disagreed that their medical school curriculum had sufficiently prepared them to manage pain (Figure 1).

Over half of the respondents disagreed or strongly disagreed that they were adequately prepared to manage chronic pain, cancer-related pain, and complicated cases of pain. Specifically, respondents did not feel prepared to treat patients dependent on narcotics, assess pain in patients with impaired cognition, distinguish between and treat neuropathic and nociceptive pain, assess and manage pain associated with cancer, or manage pain at the end of life. Modalities in which respondents felt sub-optimally prepared included physical rehabilitation, behavioral therapy, non-opioid pharmacologic modalities, and alternative medicine approaches for pain management. Other areas of perceived inadequate preparation included creating comprehensive interdisciplinary management plans for chronic pain, knowing when to refer to a pain clinic, managing pain in pediatrics, and managing pain in the elderly.

Notably, 90% of respondents did not feel adequately prepared to order/prescribe intravenous opiates, order/prescribe oral narcotics, perform opioid dosage conversion between the oral and IV routes, or understand state and federal requirements for prescribing opioids.

Over half of the respondents agreed or strongly agreed that their medical school adequately prepared them in the competencies of basic pain physiology and basic assessment of pain and narcotics-related complaints. This included the ability to assess acute pain, assess chronic pain, compassionately discuss pain with patients, understand the physiology and pharmacology of pain, understand addiction's role in pain management, identify factitious disorders and drug-seeking behavior, assess and manage postoperative pain, know when to seek surgical and interventional treatments for pain, know how to interpret urine drug screening tests, recognize a patient who has overdosed on narcotics, treat a patient who has overdosed on narcotics, and recognize a patient with an opioid use disorder.

When asked about specific experiences, two respondents had rotation experience in a pain clinic and four had simulation cases on pain management during medical school; however, none of those respondents felt the experiences were useful. Notably, 79.0% of residents said they disagree or strongly disagree that their medical school helped prepare them to manage and treat chronic pain, in particular.

## Discussion

Fifty million adults in the United States meet criteria for the diagnosis of chronic pain, and an estimated two million adults currently abuse pain medications [3,5]. This condition has gained national attention because of the burden imposed on both patients and society. Historically, suboptimal practices, such as overprescribing opioids and inadequate monitoring of iatrogenic addiction, have contributed to this problem [11]. If any progress is to be made to decrease pain-related morbidity and to improve pain management practices, it is imperative to examine how future physicians are being trained in managing pain.

A recent study by Howley et al. set out to identify how medical schools are tackling this issue and what systems are currently in place to prepare medical students to address the opioid epidemic [7]. They conducted a 21-question survey, completed by 102 medical school curriculum deans, to see how many programs were addressing four domains of pain education. These domains included: 1) the nature of pain, 2) pain assessment, including assessment of substance use disorder (SUD), 3) pain management, including treatment of SUD and opioid overdose, and 4) the context of SUD and pain. Eighty-seven percent of respondents reported addressing all four, with 100% of respondents addressing at least two. This study also highlighted three challenges to pain education, which included a lack of faculty expertise in the field, limited time in the curriculum, and difficulty assessing knowledge of pain. With this last challenge, respondents specifically cited a lack of standardized measures to assess knowledge of pain.

Another study by Ung et al. identified studies that have assessed medical and nursing student knowledge of pain management, and Patel et al. conducted a similar study to assess pharmacists’ knowledge of pain management [4,9]. Ung et al. found that the KASRP has been used to assess knowledge of pain management in healthcare professionals, notably in nine investigations studying nursing student competency in the United States, United Kingdom, Israel, and Jordan [4]. One study by Sloan et al., which assessed medical students’ cancer pain management knowledge with an Objective Structured Clinical Examination following intervention with a Structured Clinical Instruction Module showed a statistically significant improvement following the intervention [12]. To date, however, resident knowledge of pain and pain management has not been studied with the KASRP or with the intent to highlight areas for improvement in medical school curricula.

This study has several limitations, including small sample size, and only using first-year residents in internal medicine at a single residency program. We surveyed these residents in their second or third month of training and, as such, these trainees may not have had adequate experience in managing pain to make judgments about whether they learned enough in medical school. Further studies should follow the progress of these residents and stratify scores by years of residency. Additionally, it would be beneficial to include residents from other specialties such as surgery or emergency medicine. Ultimately, doing more extensive multi-institution trials are needed to validate these findings. Future studies should also stratify knowledge of pain management by the type of undergraduate medical curriculum (allopathic, osteopathic, foreign) and by the setting of the medical school (urban, rural, community-based) to identify other factors that may contribute to the understanding of pain management.

## Conclusions

Chronic pain is a widespread problem with complex mechanisms. As such, it is vitally important that medical schools assess the efficacy of their pain curricula and amend it accordingly to ensure that future physicians are properly equipped to address such a pervasive and complex disease process. In this study, internal medicine residents demonstrated the least proficiency in specific areas, including managing cancer and postoperative patients, prescribing opioids, and mitigating the risk of adverse effects of opioids. While the residents showed proficiency in areas including basic physiology and clinical reasoning as well as cultural competency and ethics, it indeed does require a broader knowledge of chronic pain, subtypes of pain, as well as more detailed instruction on opioids and the risks associated with them. Combining low knowledge scores with dissatisfaction about preparation at the medical school level indicates that improvements can occur at the medical school curricular level to better train physicians on how to best manage pain in an era that demands more expertise in this area. We hope this study will stimulate discussion regarding the value of critically assessing resident knowledge of pain management in order to target areas of improvement in the curricula of medical schools.
